# “Still learning and evolving in our approaches”: patient and stakeholder engagement among Canadian community-based primary health care researchers

**DOI:** 10.1186/s40900-018-0132-0

**Published:** 2018-12-03

**Authors:** Claire Kendall, Michael Fitzgerald, Rachel Seoyeon Kang, Sabrina T. Wong, Alan Katz, Martin Fortin, Emilie Dionne, Kerry Kuluski, Mary Ann O’Brien, Jenny Ploeg, Lois Crowe, Clare Liddy

**Affiliations:** 1 C.T. Lamont Primary Health Care Research Centre, Bruyère Research Institute, Ottawa, ON Canada; 2 Department of Family Medicine, University of Ottawa, Ottawa, ON Canada; 3 Institute for Clinical Evaluative Sciences (IC/ES), Toronto, ON Canada; 4 Li Ka Shing Knowledge Institute, St. Michael’s Hospital, Toronto, ON Canada; 5 Ottawa Hospital Research Institute, Ottawa, ON Canada; 6 School of Medicine, University of Ottawa, Ottawa, ON Canada; 7 School of Nursing, University of British Columbia, Vancouver, BC Canada; 8 Centre for Health Services and Policy Research, University of British Columbia, Vancouver, BC Canada; 9 Manitoba Centre for Health Policy, Rady Faculty of Health Sciences, University of Manitoba, Winnipeg, MB Canada; 10 Department of Family Medicine, Rady Faculty of Health Sciences, University of Manitoba, Winnipeg, MB Canada; 11 Department of Community Health Sciences, Rady Faculty of Health Sciences, University of Manitoba, Winnipeg, MB Canada; 12 Department of Family Medicine and Emergency Medicine, Université de Sherbrooke, Chicoutimi, QC Canada; 13 Centre Intégré Universitaire de Santé et de Services Sociaux du Saguenay-Lac St-Jean, Chicoutimi, QC Canada; 14 St. Mary’s Research Centre & Department of Family Medicine, McGill University, Montreal, QC Canada; 15 Bridgepoint Collaboratory of the Lunenfeld-Tanenbaum Research Institute, Sinai Health System, Toronto, ON Canada; 16 Institute of Health Policy, Management and Evaluation, Dalla Lana School of Public Health, University of Toronto, Toronto, ON Canada; 17 Department of Family and Community Medicine, University of Toronto, Toronto, ON Canada; 18 School of Nursing, Faculty of Health Sciences, McMaster University, Hamilton, ON Canada; 19 Department of Health, Aging and Society, Faculty of Social Sciences, McMaster University, Hamilton, ON Canada; 20 McMaster Institute for Research on Aging, McMaster University, Hamilton, ON Canada; 21 Aging, Community and Health Research Unit, McMaster University, Hamilton, ON Canada; 22 Department of Family Medicine, Faculty of Medicine, University of Ottawa, Ottawa, ON Canada

**Keywords:** Patient engagement, Stakeholder engagement, Community based, Primary care, Canada

## Abstract

**Plain English summary:**

Increasingly, health researchers are conducting their research in partnership with non-researchers such as patients and caregivers, advocacy groups, clinicians, and policymakers. The idea behind this partnership is to make research more relevant and appropriate. However, so far there is not much evidence about how this partnership or engagement actually affects research. We conducted an online survey of 12 teams in Canada that have engaged patients and other stakeholders in community based health research, partly as a requirement to obtain funding. We found that in many cases, the teams have engaged a wide variety and large number of stakeholders, and have involved them in many different stages of their research. Teams reported that their overall experience of this approach to research has been positive, but some challenges have been encountered along the way. Some teams found that it was difficult to communicate appropriately with all the stakeholders, and to keep them informed when research was going slowly. Other teams had trouble finding government representatives to work with. Several teams noted that engagement is time-consuming, and requires a lot of effort. Nevertheless, all teams reported that they had learned from the experience, and found it valuable. As a result, Canadian health care researchers are better positioned to engage with patients and other stakeholders in the future.

**Abstract:**

**Background**

Patient and other stakeholder engagement in research is increasingly important, but there is limited evidence of its impact. In 2013, the Canadian Institutes of Health Research launched a five-year Community Based Primary Health Care (CBPHC) initiative that funded 12 teams for innovative approaches to primary health care involving engagement with patients, communities, decision-makers, and clinicians across jurisdictions in Canada. The present study examines the extent of engagement by these teams, and the factors that affected it, either as challenges or opportunities.

**Methods**

We conducted a cross-sectional web-based survey across the 12 CBPHC Innovation Teams, in which we were also participants. We used a data collection tool developed by the Patient Centered Outcomes Research Institute that included both closed and open-ended questions.

**Results**

The quantitative data showed that the CBPHC Innovation teams have engaged with diverse stakeholders at different levels and in different stages of research. Almost all teams surveyed engaged with policymakers, most with clinicians and health system representatives, and more than half with patients, mostly at the level of consultation or collaboration. There were very few instances of stakeholder-led research reported. There was a near universal recognition of the importance of communications processes/tools in facilitating engagement, whereas time was the most commonly identified challenge. In almost all cases, challenges encountered were partially if not fully resolved.

The qualitative findings showed that each team’s engagement was contextualized by factors such as the jurisdictions and geographic scope of the project, the number and type of stakeholders engaged and their level of involvement. These intersected with the researchers’ motivations for engagement, to give rise to diverse experiences, but ones that the CBPHC teams assessed positively as an approach to research.

**Conclusions**

Over the past five years, primary health care researchers in Canada have been actively engaging with patients and other stakeholders. The wide range, extent and nature of that engagement shows that these researchers have anticipated developments in this approach to research and are thus in a position to support and strengthen future efforts to understand the impact of this engagement on health care outcomes.

## Background

The role of patient and other stakeholder engagement or involvement in enhancing the relevance and impact of research findings has become an important criterion in health research funding decisions [1, 2]. (See Table 1 for how “patient” and “stakeholder” are used in this article).

**Table 1:** A note on terminology

The term “patient” is used in different ways in the literature, which seems to depend on region. In the North American context, it is often used in an inclusive sense, comprising patients and family members/caregivers (e.g., Canadian Institutes of Health Research (CIHR) [57]), and sometimes patient advocacy organizations (e.g., Patient-Centered Outcomes Research Institute (PCORI) [58]). In this paper, however, we use “patient” in the exclusive sense, as the survey instrument we used identifies patients, family members/caregivers and patient advocacy organizations separately. Throughout, we use “stakeholder” in CIHR’s sense (“An individual, group or organization having a “stake” in an issue and its outcome.” [59]) We use the phrase “patients and other stakeholders” to reflect these distinctions.	

While stakeholders such as patients, caregivers, clinicians and policymakers have often been considered “passive audiences for research results” [3], researchers have increasingly sought their input across the range of research activities, from agenda setting to knowledge translation and dissemination [4–7]. The nature of such engagement varies from (sometimes tokenistic) co-option to collective action or full partnership in which stakeholders lead the research [8]. A number of research approaches have embraced an expanded stakeholder role, including participatory research [8–10], comparative effectiveness research [3, 11–13], implementation research [14], patient-oriented research [5, 15–17] and patient-centered outcomes research [3, 4, 11, 13, 18–20].

Over the past two decades health services, ministries and funding agencies have taken efforts to promote such engagement [2, 21–25]. As Domecq et al. point out, “there is a growing consensus about the crucial role of patient involvement in research, which may improve the value of healthcare research” [7]. Despite this growing interest, relatively few studies have evaluated the process and outcomes of patient and other stakeholder engagement [19, 26–30], particularly in the Canadian context [23], an issue attributed to poor reporting of engagement strategies in published research [31], particularly of researchers’ own expectations [32, 33], and the lack of a formal or systematic process to characterize or evaluate engagement [4, 7, 23, 29, 32, 34]. The difficulty in producing evidence of impact has also been attributed to the “focus on narrowly defined interventions” in the health literature that fails to take into account the more extended effects of engagement [35], and to the tendency to view engagement as an intervention in the research process itself [36, 37].

Some studies suggest it may be more productive to focus primarily on researchers’ expectations of engagement [22, 32]. However, many research studies report their engagement activities inadequately [38], making it difficult to determine their impact, which is complex because it involves contextual factors and “mechanisms” such as underlying beliefs, values, attitudes, etc. [39]. Of particular importance are the values that motivate engagement [2], which the literature categorizes as *moral* or *normative* (e.g., empowerment and rights), *instrumental* or *substantive* (e.g., improving research quality), and *process* (e.g., having to do with research conduct) [2, 3, 6, 29, 40–46].

### Canadian Institutes of Health Research’s (CIHR) community based primary health care (CBPHC) innovation teams

In 2011, the Canadian Institutes of Health Research (CIHR) announced its Strategy for Patient Oriented Research (SPOR), subsequently releasing its Patient Engagement Framework in 2014 [5]. In the midst of this process, CIHR launched its Community Based Primary Health Care (CBPHC) initiative to develop and compare innovative CBPHC models across jurisdictions by supporting researchers “to conduct original research on innovative models of care delivery, build capacity for research excellence and translate evidence for uptake into practice and policy” [47]. One component of this program funded 12 CBPHC Innovation Teams with two research priorities: (i) access for vulnerable populations; and (ii) chronic disease prevention and management. The CBPHC Innovation Teams were geographically distributed across Canada and were highly diverse, ranging from one to eight principal investigators and up to 42 co-investigators for a total of 280 different researchers [48].

These teams were required to comprise investigators from at least two jurisdictions, at least two decision makers (“typically a health-system manager, policy-maker, community-based healthcare organization leader, or clinician leader” [49]) from two different jurisdictions, and at least one health professional. As part of the funding application, teams had to provide “evidence of a citizen and patient advisory process that enables the provision of feedback on proposed care delivery models, relevant targets for evaluation, community partnerships, and patient, family and community involvement” [49]. Teams were therefore required to *collaborate* with decision makers and health professionals, but only to *consult* with citizens and patients.

The present study adds to the evidence base on patient and other stakeholder engagement in the Canadian context by examining the extent of such engagement by the CBPHC Innovation Teams, and provides some insight into the way that engagement affects researchers’ understanding of their research projects, and how contextual factors and motivational values present challenges to and opportunities for successful engagement.

## Methods

### Study design

One of the 12 CBPHC Innovation Teams, in collaboration with principal investigators from other teams, conducted a cross-sectional web-based survey to assess stakeholder engagement across the 12 CBPHC Innovation Teams. We were therefore study participants as well as researchers.

### Participants

The nominated principal investigator (NPI) of each CBPHC Innovation team was asked to identify the most appropriate member of their team to complete the survey, who was then emailed the informed consent form and a link to the survey on an encrypted web platform, Hosted in Canada Surveys. Follow-up emails were sent after two and four weeks to remind participants to complete the survey. If there was no response after four weeks, we followed up with the NPI to request assistance in facilitating a response.

### Data collection

We used the Data Collection Tool developed by the Patient-Centered Outcomes Research Institute (PCORI) to assess patient and other stakeholder engagement [4], after modifying it for the Canadian primary health care context (Appendix 1). This tool includes both closed and open-ended questions. “Ethics processes” and “Research team governance” were added as response options for the stages in the research process in which stakeholders were involved, to reflect the core engagement areas outlined in CIHR’s SPOR framework [5]. Two questions under the “Challenges” section were merged to simplify the survey and take advantage of web-based delivery. Details of the tool and its development have been previously described by Forsythe et al. [4].

### Data analysis

Data were imported into Excel for cleaning and analysis. Descriptive statistical analyses of responses to categorical survey questions were used to compare and characterize engagement across the CBPHC program. Responses to open-ended survey questions were iteratively analyzed for themes and exceptions [50]. These were initially coded and grouped into themes by one author through a line by line reading; the research team then reviewed the themes for disconfirming evidence, with any discordance being resolved through discussion. Because responses to the open-ended questions were not extensive (~ 8500 words), these data were unlikely to support extensive qualitative analysis [51]. Because of the small sample size and the heterogeneity of the quantitative data, we analyzed the narrative responses to the open-ended survey questions from each team (e.g., motivations, challenges and contributions) in tandem with the descriptive characteristics of team engagement (e.g., types of stakeholders engaged, level of engagement). This allowed us to explore how engagement proceeded in the context of descriptive characteristics found in the quantitative data.

## Findings

Ten of the CBPHC teams responded to the survey; the two remaining teams declined to participate. Surveys were completed between July 2017 and January 2018, in the final year of the CBPHC funding initiative.

### Quantitative survey responses

#### Stakeholders

**Fig. 1:**
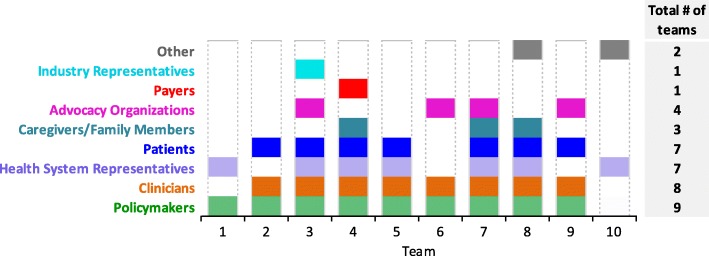
Stakeholders engaged by CBPHC teams (*n* = 10)

As Fig. 1 shows, all respondents reported engaging patients or other stakeholders in their research projects, with teams engaging between two and six different types. Seven teams engaged patients, three of which also engaged caregivers/family members and another three patient advocacy organizations. The latter were also engaged by one team that engaged neither patients or caregivers. Other commonly engaged stakeholders included policy makers (9 teams), clinicians (8), and health system representatives (7). Two teams reported engaging stakeholders not listed: (i) a volunteer community organization; and (ii) international liaisons. Only one respondent reported engaging payers, explaining that *“our policy makers were the same as payers – dual role”* (T4), and one reported engaging industry representatives. Responses to the open-ended questions suggested that in some cases respondents may have included one type of stakeholder under another, for example, clinicians and policymakers under “Health System Representatives”.

In response to the question asking *how many* of each type of stakeholder the team had engaged, five respondents indicated they had engaged more than five patients, six that they had engaged more than five clinicians, six that they had engaged more than five health system representatives, and five that they had engaged more than five policymakers. The remaining types of stakeholder were engaged in far fewer numbers, the least being industry representatives, only one of whom was engaged by one team (data not shown).

#### Nature of involvement

**Fig. 2:**
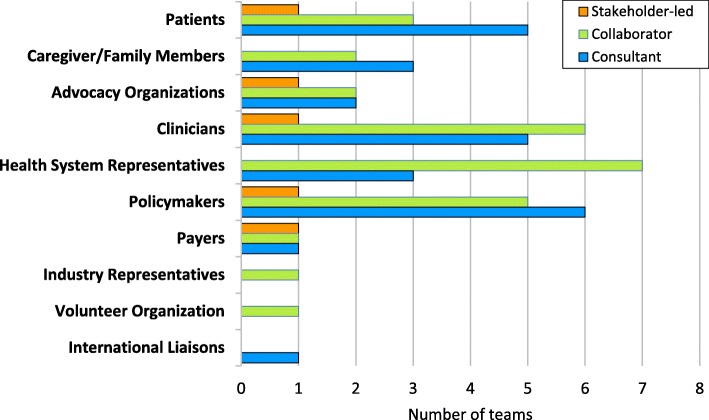
Nature of stakeholder involvement (n = 10)

As Fig. 2 shows, five teams reported consulting with patients (i.e., patients provide views on various aspects of the research), two of which also collaborated with patients (i.e., they were formally engaged on the project as ongoing partners). Another team reported engaging with patients only as collaborators. Lastly, only one team reported engaging with patients as stakeholders leading the research. This latter level of engagement, stakeholder-led research, was the least common and least frequent across all stakeholders, involving only five of the ten identified types; one team alone accounted for four of these instances. The majority of engagement was evenly divided between consultation and collaboration. Two respondents identified workshop participation as another type of involvement.

#### Stages of research

**Fig. 3:**
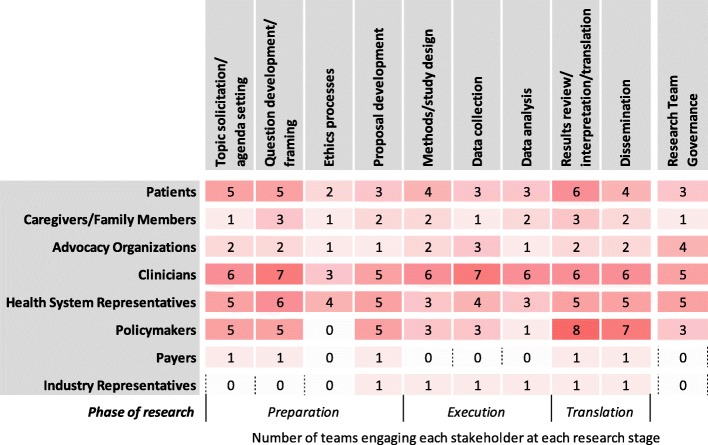
Stakeholder engagement by stage of research (n = 10)

As Fig. 3 shows, looking at the CBPHC teams as a whole, patients were engaged at all stages of research, the most common being results review/interpretation/translation (*n* = 6 teams), topic solicitation/agenda setting (*n* = 5) and question development/framing (n = 5). All teams that engaged patients did so in at least three stages of research, with one team engaging patients at ten research stages, encompassing all identified stages except for ethics processes and adding workshop participation (data not shown). An additional stage identified by another respondent was *“training for the delivery of the intervention”* (T5) (data not shown). Clinicians were the stakeholders most commonly engaged across the majority of research stages, although the largest number of teams engaged policymakers for results review/interpretation/translation. Payers and industry representatives were engaged by the fewest number of teams across all research stages. Involvement in research team governance was more common for patient advocacy organizations, clinicians and health system representatives than for other types of stakeholders.

#### Frequency of engagement

**Fig. 4:**
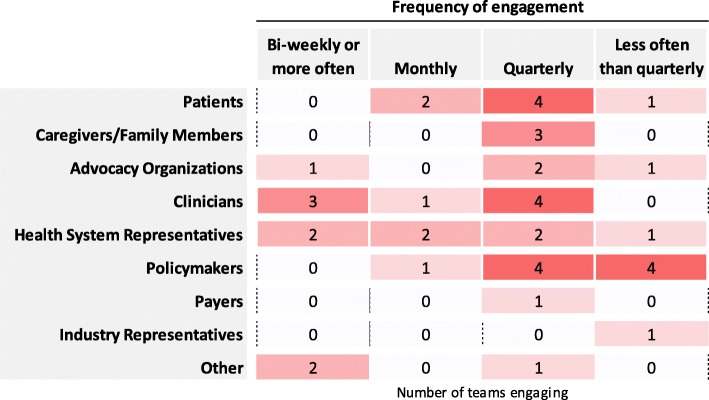
Frequency of stakeholder engagement, by number of teams

Respondents were asked about the frequency of their email, spoken and in-person contact with stakeholders. As Fig. 4 shows, CBPHC teams engaged stakeholders most commonly on a quarterly basis. More than half of engagement with patients and all engagements with caregivers/family members were on this basis. Clinicians and health system representatives, in contrast, were engaged somewhat more frequently, with three teams engaging clinicians on a biweekly or greater basis. Almost all policymakers were engaged quarterly or less often.

#### Length of engagement

Because the question about how long the respondent’s team had been working with each type of stakeholder was open-ended, many of the responses were ranges, multiple lengths, or were vague, which made it difficult to do any precise analysis of the responses. Overall, the data include 11 indications of engagement pre-dating the grant, 17 where it was co-terminous, and 28 where it began sometime after (data not shown).

#### Facilitators of engagement

**Fig. 5:**
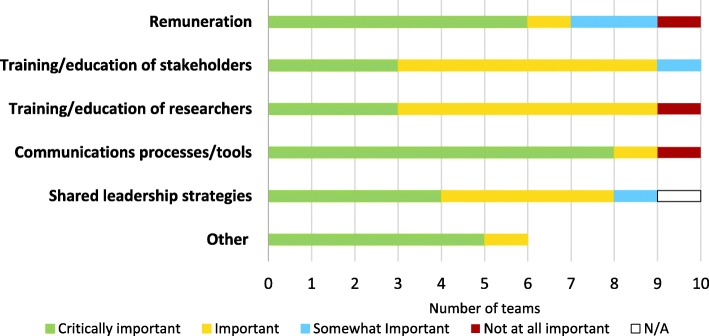
Evaluation of facilitators to engagement (n = 10)

Respondents were asked a global question using a four-point Likert scale about the importance of each of a provided list of facilitators of engagement. As Fig. 5 shows, the most important facilitator was communications processes/tools, identified as “Critically important” by eight respondents and as “Important” by one. Remuneration was also identified as “Critically important” by a majority of respondents. Very few respondents identified any of the facilitators as “Not at all important”. Other facilitators respondents used were the *“Research Manager,” “spending time in small group and individual interactions”* (T4), *“research governance of grant”* (T8) and *“having a structured approach”* (T9).

#### Challenges to engagement

**Fig. 6:**
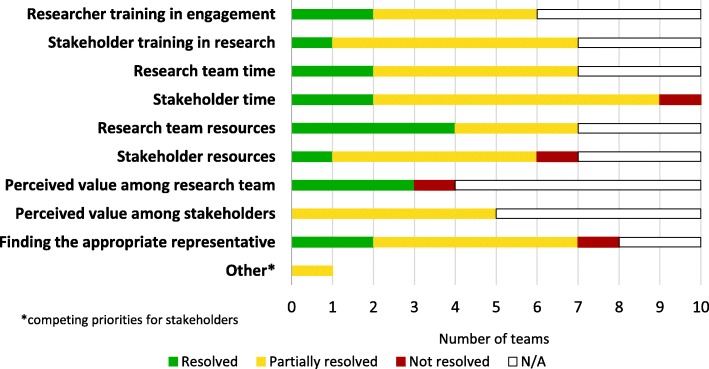
Challenges to engagement (n = 10)

Respondents were asked a global question using a three-point Likert scale about the extent to which each of a provided list of challenges was resolved. As Fig. 6 shows, the most commonly identified challenge was stakeholder time, which seven respondents indicated was “Partially resolved,” two that it was “Fully resolved,” and one “Not resolved.” Challenges were identified as “Partially resolved” in about two-thirds of the cases, and “Fully resolved” in about a quarter. One respondent identified “*competing priorities*” (T8) as an additional challenge.

#### Summary

In summary, the quantitative data show that CBPHC Innovation teams have engaged with diverse stakeholders at different levels and in different research stages. Amongst the few common characteristics were a near universal engagement with policymakers, particularly in the Translation phase of research, and a near universal recognition of the importance of communications processes/tools in facilitating patient and stakeholder engagement.

### Qualitative findings

#### Relationships

The qualitative data clearly indicated both the instrumental values (e.g., as a facilitator for finding appropriate representatives) and ethical values (e.g., *“Nothing about us, without us”* (T9)) that motivated teams’ engagement. Almost all teams indicated that they had an existing relationship with one type of stakeholder or another, and some referred to *“existing contacts”* (T5), *“existing networks”* (T9) or *“other relationships”* (T8) as ways in which they established relationships with other stakeholders.

Stakeholder engagement took place at the macro (organizations representing communities), meso (communities or groups of individuals) and micro (individual) levels. This was notably the case for one team that was morally motivated to engage with Indigenous communities. *“Long standing relationships”* (T1) with an Indigenous organization allowed these researchers to collaborate with appropriate health system representatives in those communities. In particular, this was facilitated by the Research Manager who was *“housed”* at the Indigenous organization: *“This position was critically important in developing the initial relationship and facilitating communication between the academic and community research teams”* (T1). The importance of this position and the prior relationship is evident from the challenge this team encountered with *“establishing relationships and obtaining approval*” (T1) in the *non*-Indigenous communities, where *“knowing who to contact, was an issue*” (T1) and there was a lack of “*coordinated effort from leadership in the [jurisdiction’s] health system”* (T1). For this team, *“effective and clear communication between all stakeholders and team members”* (T1) was essential in addressing such challenges.

Relationships themselves could also prove to be a challenge, particular with policymakers, due to the different lifecycles of policy-making and health care research (see below).

#### Communication

Consistent with the quantitative data, almost all respondents considered communication to be a critically important facilitator of engagement, but responses to the open-ended questions suggest that this was contextually specific. For example, a team that engaged with six different types of stakeholders indicated a difference between communication with patients and caregivers on the one hand, and health system representatives, payers and policymakers on the other. The patient and caregiver engagement was at both the consultant and collaborator levels, and was motivated moralistically *“to ensure that patients and caregivers had a voice at the table”* (T4). Engagement occurred on a quarterly basis, and did not include the Execution phase of research. Although the relationships pre-dated the CBPHC grant, and no particular challenges to this engagement were reported, the researchers noted that they needed to use *“a different less technical and more approachable language”* (T4). More significantly, they also suggested that communication needs run in both directions, as engagement with patients *“may require extensive socialization and discussion with researcher[s], many of whom are not used to considering the advisory input of patients in agenda setting or decision-making within the research team”* (T4). The contribution that resulted, however, was *“to lead us to ask questions in ways that are more directly relevant to improving navigation in the health system from the patient’s perspective as opposed to from the provider perspective”* (T4). Another team indicated that the change in the language they used was motivated by patients: “*We agreed on term of references for the use of certain words during our meeting that were judged offending (patients with multi morbidity do not want to be called multi morbid!)”* (T2).

The motivations for T4’s engagement with decision-makers were process (*“First is the requirements of the competition to have these representatives”* (T4)) and instrumental (*“to have a shorter line to actual health system impact”* (T4)). These relationships also pre-dated the CBPHC grant. Engagement occurred quarterly and did not include the Execution phase of research. The challenge of incorporating the interests of decision makers as collaborators and consultants involved paying attention to their *own* communication needs: “*Extensive discussion and true listening to the ways that policy makers and decision-makers communicate with their audience to determine how we could and should present our key findings”* (T4). From this, the researchers learned that *“engaging with system decision-makers must appreciate the simplicity of the language that they need to communicate with their stakeholders and the lack of tolerance for extensive nuance”* (T4) and identified as critically important the facilitator of *“spending time in small group and individual interactions developing trust and relationships with key members of the team”* (T4). In this context, one of the important contributions was relationship building: *“[they] have lead us to spend time to work with them to listen to their questions and develop longer term relationships”* (T4).

#### Diverse populations and communities

Relationship building and communication were also factors in a second theme evident in the qualitative data, namely, the challenge of engaging diverse communities. Another team that engaged six different types of stakeholders learned that engaging with patients and populations involves relationship building not only with patients themselves, but also with their communities:

*“it is vital to reach out first to community members and workers who are well accustomed with these patients and these communities, if relevant; they are the ones who can support making contact, translating our different language (e.g., as researchers, we tend to use a terminology that is not intuitive to the general public, even less to vulnerable populations, and which can be a ‘turn off’ for them…). We needed to invest in building relationships with the patients and within the communities.”* (T5)

This team engaged with more than five patients, some of whom they had a relationship with that pre-dated the grant, at the level of consultant in the preparatory phase of the research. The instrumental motivations for this engagement were *“effectiveness of outcomes…it is necessary that we connect with these members, to understand well their needs, perspectives, values, etc.”* (T5). Despite the pre-existing relationship, the team found that they *“needed more clear guidelines of what was required for them. The patients also needed to trust those at the details, and understand well what their role as partner was, and how they were experts in their field”* (T5).

This team also highlighted a procedurally important aspect of communication, namely consistency: *“Developing and using consistency in communication mechanisms is key. Using different communication strategies as well”* (T5). Despite these challenges, one of the contributions identified was that *“participatory action research creates sustainable partnership, rich and innovative, diverse, communities of exchange and partners that survive and go beyond the particularity of our research programme”* (T5).

Another team that involved all stakeholders in the majority of research stages including research team governance engaged with patients as collaborators out of moral motivations (“*to ensure patient perspectives are represented”* (T7)) and instrumental motivations (*“that the research is relevant”* (T7)), and recruited them *“from our initial studies”* (T7). The engagement began after the grant was awarded and occurred on a quarterly basis. This team encountered challenges with *“role clarity and supporting previous research participants/subjects in understanding their new advisory/collaborator roles”* (T7)*,* which also pertained to engagement with caregivers/family members. Addressing these challenges meant that the researchers *“needed…to learn how to tailor approaches to ensure they would align with our patients and family caregivers’ knowledge and interest”* (T7), on the one hand, but also *“to be aware of and acknowledge their changing health status”* (T7). From these and the other stakeholders, however, researchers learned that *“they each add a unique perspective to the research and add value in their lived experiences, knowledge and skills”* (T7). Because of the high level of engagement of many stakeholders across research phases, the team also noted that *“it is time consuming to keep all these stakeholders engaged in meaningful ways”* (T7).

#### Time

Lack of time and changing schedules were a challenge noted by almost all teams, and did not seem to depend on the number of stakeholders engaged. For example, one team that engaged with advocacy organizations at the consultant level, clinicians as collaborators, and policymakers as collaborators and consultants for both instrumental reasons (*“Their knowledge of the subject, The clinician’s primary health care experience and his research experience”* (T6)) and process reasons (“*their local networks”* (T6)), noted challenges in *“establishing a time for regular contact”* (T6) with the advocacy organization and in *“finding time to meet”* (T6) with the policymakers. The initial learning in this case was *“be flexible to meeting times, go where they are”* (T6).

In contrast, time and scheduling challenges in engaging with clinicians were mentioned by several teams. For example, a team that engaged six different types of stakeholders found that in engaging clinicians at the levels of consultant and collaborator for instrumental reasons, *“maintaining engagement was challenging due to clinicians’ time constraints”* (T3), which this team did not encounter with any of the other stakeholders. Nevertheless, the learning reported was that *“engagement takes time, but it’s worth it”* (T3). A different team that engaged with three types of stakeholders found that the challenge of engagement with clinicians as consultants was that they are *“busy people, difficult to meet with*” (T2).

The only team to indicate that all of its engagement (with patients, advocacy organizations, clinicians and policymakers) was stakeholder-led also noted that scheduling was *“the key challenge”* (T9) in engaging with clinicians, even though *“family physicians were the driving force behind this project, and as such, formed an integral part of the team”* (T9) and the researchers *“recruited specialists who have also been engaged since the beginning”* (T9). The engagement was instrumentally motivated (*“understanding the clinician needs and advancing knowledge to expand, enhance or change service delivery models”* (T9)) and occurred more often than biweekly. For this team, however, engagement with policymakers *“was the most challenging part of the grant in many ways”*(T9). These stakeholders were engaged with from the beginning of the grant for instrumental reasons (*“We anticipated that having engaged policy makers would have led to a higher chance of innovations arising from the grant succeeding”* (T9)), although the frequency of engagement was low, being less often than quarterly. The researchers found it difficult to know *“which policy makers to reach out to”* (T9) and learned that they need *“to find better ways to engage policy makers”* (T9).

#### Bureaucratic context

For several teams, difficulty in finding the right policymakers to engage with appears to have been exacerbated by personnel turnover (*“policymakers changed in each of our partner provinces within the first few years of funding”* (T9), *“dealing with change in leadership and change in position”* (T5)) and bureaucratic structures (*“We were challenged by the many layers and structures within government and had to take time to learn its organizational structure…Another challenge is the turnover of policy makers and identifying the right people to discuss our research with”* (T8)). Commitment and accountability was another challenge in engaging with policymakers. For example, a team that only engaged with two stakeholders found that with policymakers, “*time commitment, scheduling of meetings and follow-through with the recommendations”* (T1) were all challenges, and that *“often the decision makers at the table were those that had very little decision making power”* (T1). Finally, the cross-jurisdictional nature of the CBPHC grant created challenges in *“dealing with policy makers across Canada (had to determine who they were)”* (T8).

#### Effects of engagement

Almost all teams indicated either having a plan to assess the influence of patients and other stakeholders on the research, or an interest in doing so. However, few provided any concrete details of such plans. Similarly, most teams had either collected or were planning to collect information regarding the research experiences of patients and other stakeholders through a variety of means, such as *“interviews, self-assessment, and focus group data”* (T5). One team had obtained funding *“to survey and interview our stakeholders in their experiences”* (T7), whereas two other teams (T4 and T9) had published papers on their patient and stakeholder experiences.

The teams’ “initial learnings” tended to focus on what the researchers themselves had learned about patient and other stakeholder engagement, and in large part overlapped with the engagement challenges they faced. However, several teams mentioned the effects of engagement on the organizational and governance aspects of their research: *“Patients are important to reflect on exactly what is going on in primary care. We as clinician or researchers have a vision that is biased so we need their input to stay on track”* (T2); *“Being open to different ideas is important, even though it sometimes leads you to change the direction of the work”* (T3); *“governance structure (input of stakeholders built right in to the grant); stakeholder input in leadership committee”* (T8). One team pointed out that this level of involvement also had an impact on stakeholders: “*implementation is likely to be more successful if they…are a real collaborator with responsibilities”* (T2).

The impact of engagement on the research itself was more evident in the context of “the most significant contributions” by patients and other stakeholders, predominantly in terms of relevance. The most detailed response contrasted the contributions of the different stakeholders. The *“patient and caregiver”* (T4) led the team *“to ask questions in ways that are more directly relevant”* (T4), whereas the contributions of decision-makers were an *“opportunity to answer the questions that they are prepared to act upon”* (T4) and led the team *“to try to develop early advice from existing evidence*” (T4). In each of these ways, then, the team ended up pursuing its research differently than would otherwise have been the case.

## Discussion

This study examined how Canadian primary health care researchers have perceived their engagement with patients and other stakeholders in the course of CIHR’s five-year funding initiative for 12 CBPHC Innovation Teams. The findings show the complex and multi-faceted nature of patient and other stakeholder engagement in these projects. Respondents indicated that they encountered a number of challenges in the course of their engagement activities, such as communication, time, and even finding appropriate stakeholder representatives with whom to engage. Nevertheless, the responses also show that researchers have engaged patients and other stakeholders in a way that closely aligns with the goals articulated in CIHR’s SPOR Patient Engagement Framework [5]. The challenges respondents outlined regarding this engagement, and the efforts teams undertook to clarify roles and expectations, communicate effectively, and be flexible and adaptable certainly indicate that the interaction has been active, and suggest that it has also been meaningful, at least for the researchers. Although only infrequently mentioned by respondents, the funding requirement that CBPHC teams comprise both decision-makers and clinicians, and that they consult with patients, undoubtedly had an influence on the nature and level of their engagement. Interestingly, however, the majority of the teams that reported engaging patients went beyond the CIHR-mandated level, to actively collaborate with these stakeholders *throughout* the research process, rather than simply seeking feedback on aspects of the research.

This study adds to the evidence base for patient and other stakeholder engagement in the Canadian context, and particularly to our knowledge about the processes of engagement. For example, the motivations these researchers indicated for engaging different stakeholders seem to correspond well to the three types of values – moral or ethical, instrumental or substantive, and process – that have been reported in other studies [6, 26, 43, 46]. But the responses also show how these motivations cut across stakeholder groups, levels of engagement, and stage of research, which highlights the contextual nature of engagement. These findings also provide some insight into how the actual processes of engagement affect the progression of research projects. For example, finding appropriate representatives of different types of stakeholders with whom to engage had an effect on project timelines, which in turn had repercussions for engagement. The difficulty many of the CBPHC teams had in this regard with policymakers suggests that researchers need to take bureaucratic context(s) into account when developing projects incorporating such engagement.

The highly contextual nature of engagement can also be seen in the effect this had on communication, another key issue pointed to in earlier studies [23, 52–54]. Findings from the CBPHC teams provide further evidence of the centrality of communication in effective engagement, but this was realized differently for different stakeholders at different levels of involvement, and possibly at different stages of research. For example, with patients, the issue of communication revolved around the use of non-technical, lay language and sensitivity to vulnerability. With other stakeholders such as health care representatives and policymakers, the communication challenge concerned ways of aligning with their needs and interests. These findings suggest that stakeholders’ interests may also vary. For example, patients’ interests arise out of lived experience, whereas health care representatives’ interests could be process or instrumental. Interestingly, the findings also suggest that policymakers’ interests may be much less specific, possibly because of competing priorities. If this is so, it adds another challenging aspect to engaging such stakeholders.

In terms of the implications for further research, a significant finding was that very little of the engagement involved stakeholders *leading* the research. Although the reasons for this were not probed by the survey questions, the responses as a whole suggest that lack of time, support and mechanisms to build relationships could be factors. This suggests a possible need for more discussion in the Canadian primary health care research community about the optimal prevalence of stakeholder-led research. The findings also suggest that one of the ways that engagement with patients and other stakeholders works is by deepening researchers’ understanding of how their research is relevant to diverse audiences. Such findings support the idea that evaluations to date may be overlooking a key mechanism in the impact of engagement on research, namely researchers’ learning. Because patient and other stakeholder engagement is complex and “takes many forms and operates at many different levels” [32], more attention needs to be paid to the contexts and mechanisms of engagement to understand its wider impacts [22, 33, 55, 56].

Because the CBPHC projects are in their final stages, it is too early to evaluate the impact of engagement on primary health care outcomes, and their heterogeneity means that it is difficult to generalize from these researchers’ experiences. However, the formative role that patient and other stakeholder engagement has played in these projects can be seen in the survey responses, and is perhaps most evident in the way it has occasioned researchers’ re-evaluation and re-configuration of the various processes involved to take into account stakeholder needs and interests, for example, in communicating with vulnerable patients and accommodating policymakers’ practical requirements. Despite the novelty of this type of engagement for Canadian primary care researchers, the range and diversity of the CBPHC teams’ engagement experience evident in the survey responses demonstrate the extent to which these researchers have embraced this approach, and are now better positioned to actively collaborate with patients, decision-makers and other stakeholders in future research.

### Limitations

Although the CBPHC Innovation teams that responded to the survey represent a significant proportion of primary health care researchers in Canada, it is not known to what extent the responses are reflective of all team members, as no data were collected on the process teams actually used to complete the survey. For example, it is unknown whether survey responses included direct feedback from patients and other stakeholders, particularly their views of the various stages of the research. The structure of the survey tool did not allow for response to all questions by type of stakeholder, which may have led to underreporting in some cases, and possible over-representation in cases where pre-formulated responses were provided. Furthermore, it should be noted that the priorities of the CBPHC project were access to care for vulnerable populations and chronic disease prevention and management, and therefore the findings of the present study may not be generalizable to all primary health care research in Canada. Finally, the PCORI study for which this survey tool was developed was aimed at a different context, namely 50 U.S. pilot projects on methods for patient-centered outcomes research [4] with the survey being conducted in the first year of a two year grant. Therefore, it did not include questions about certain factors of relevance to the CBPHC Innovation Teams, such as whether engagement that preceded the funding award had an impact on research design and execution, and the length of time required to build relationships capable of supporting engagement in research. Given these differences, and the small dataset in the present study, we felt that a comparative analysis would be problematic and therefore was not done.

## Conclusion

Over the past five years, primary health care researchers in Canada have been actively pursuing patient and other stakeholder engagement. Although this was motivated in part by requirements stipulated by the funding agency, the wide range of stakeholders, extent and nature of that engagement shows that these researchers have anticipated developments in this area and are thus in a position to support and strengthen future efforts to understand the impact of patient and other stakeholder engagement on health care outcomes.

## Appendix 1

### Data collection tool

As part of our commitment to the Canadian Institutes of Health Research (CIHR) to support cross-team collaboration efforts, we are asking that you complete this PCORI (Patient-Centered Outcomes Research Institute) survey on behalf of your CIHR’s Community Based Primary Health Care (CBPHC) team. The collected data will be used to assess patient and stakeholder engagement across the 12 teams.

This survey assesses:

- Stakeholders engaged, inclusive of patients, families, communities and organizations;
- Level of engagement (consultation, collaboration, stakeholder-led);
- Nature of relationships with stakeholders;
- Stages of the research project in which patients were engaged;
- Facilitators of and challenges for engagement.

This survey also includes open-ended questions about the above issues and lessons learned about engagement throughout the process.

We estimate the survey should take approximately one hour to complete, with the possibility of needing additional time to consult with others to complete the questions. We encourage you to discuss the questions with your team members and patient participants, to ensure that the answers are as representative as possible.

Please read over the participant informed consent form before starting this survey. Completion of this survey implies that you have consented to participate in our research study.

If you have any questions about this study, please contact Dr. Claire Kendall at 613–562-6262, Ext. 2941 or the Research Manager, Lois Crowe at 613–562-6262, Ext. 2929.

1. At this point in your CBPHC research project (s), have you engaged patients or other stakeholders in your project (s) in ways *other* than as research subjects?

- Yes
- No *(skip to Q 24)*

2. Which of the following stakeholder communities have you engaged in your CBPHC project (s) in ways *other* than as research subjects? Please select all that apply, but only one category per stakeholder engaged (i.e. please select the community with which a given stakeholder would most closely identify for the purposes of your project).

- Patient/Consumer (unaffiliated individual)
- Caregiver/Family Member of Patient (unaffiliated individual)
- Patient/Consumer/Caregiver Advocacy Organization
- Clinician
- Clinic/Hospital/Health System Representative
- Payer
- Industry Representative
- Policy Maker
- Other (Please describe)

3. a. For each type of stakeholder engaged in your project (s), please indicate the nature of their involvement. Check all that apply.

**Table:**

	Stakeholder-led^1^	Collaborator^2^	Consultant^3^	Other
<insert stakeholder>				
<insert stakeholder>				
<insert stakeholder>				

^1^Stakeholder-led research: stakeholder (s) design and undertake the research and researchers are invited to participate at the invitation of the stakeholder (s).

^2^Collaborators have an ongoing partnership/affiliation with the researchers and healthcare professionals in the research process, and are formally engaged to complete a specific research project, and therefore have greater ownership of the project (e.g., steering committee, co-investigator).

^3^Consultants are asked about their views on various aspects of the research and researchers use their views to influence decision-making regarding research (e.g., focus groups, interviews, requests for input). Consultation allows the researcher to obtain views’ without necessarily being committed to act on them.

b. Please explain the “other” ways in which stakeholders were involved in your project (s).

*For each type of stakeholder selected, the following questions will be presented (the term “patient/consumer (unaffiliated individual)” is used as an example below, but other stakeholder types might include those specified in Q 2 above). The full set of questions (4 thru 11) will be asked for each selected stakeholder type.*

You have indicated that you have engaged <insert stakeholder> in your project (s) in ways other than as research subjects. Please answer the following questions regarding <insert stakeholder> engagement.

4. How many <insert stakeholder> have you engaged in your project (s) thus far?

- 1
- 2
- 3
- 4
- 5
- More than 5

5. How was the relationship with this/these <insert stakeholder> established?
6. For how long have you been working with this/these <insert stakeholder> (on this or other research projects)?
7. What challenges did you encounter in establishing the relationship with this/these <insert stakeholder>?
8. Please describe any efforts made by your team to overcome these challenges.
9. What was your research team’s primary motivation for engaging this/these <insert stakeholder> in your research project (s)?
10. Up to this point in your research project (s), in what stage (s) in the research process have you worked with this/these <insert stakeholder>? Select all that apply.

- Topic solicitation/agenda setting
- Question development/framing
- Ethics processes
- Proposal development
- Methods/study design
- Data collection
- Data analysis
- Results review/interpretation/translation
- Dissemination
- Research team governance
- Other (please specify)

11. Up to this point, how often have you or other members of your research team emailed, spoken to, and/or met with (in person) this/these <insert stakeholder> about the project (s)?

- Not at all
- Fewer than 4 times a year
- Once every 2–3 months
- Approximately once a month
- 2–3 times a month
- More than 3 times a month

12. Based on your interactions with all of these stakeholders up to this point, what initial learnings can you offer to others regarding engaging patients and other stakeholders in research?
13. Up to this point, what are the most significant contribution(s) made by these patients and other stakeholders?

Facilitators

14. Please rate the importance of each of the following facilitators of engagement of patients or other stakeholders in research for your project (s). If the facilitator was not used by your team, please select the “N/A” option. If you have no other facilitators to add, please select “N/A” for “Other 1,2,3”.

**Table:**

	Not at all Important	Somewhat important	Important	Critically important	N/A
Remuneration (e.g., honoraria, travel support)					
Training/education of stakeholders					
Training/education of researchers					
Communications processes / tools					
Shared leadership strategies					
Other 1 (please explain below)					
Other 2 (please explain below)					
Other 3 (please explain below)					

15. Please describe the “other” facilitators your research team used to facilitate engagement of patients or other stakeholders.

Challenges

16. Please indicate to what extent you have been able to resolve the following challenges in engaging patients and other stakeholders in your research project (s). If the challenge was not encountered by your team up to this point, please select the “N/A” option. If you have no other challenges to add, please select “N/A” for “Other 1,2,3”.

**Table:**

	Not at all resolved	Partially resolved	Completely resolved	N/A
Lack of research team training/background in engagement of patients and other stakeholders				
Lack of stakeholder training/background in research process/methods				
Lack of research team time				
Lack of stakeholder time				
Lack of research team resources				
Lack of stakeholder resources				
Lack of perceived value among research team				
Lack of perceived value among stakeholders				
difficulty in finding the appropriate representatives				
Other 1 (please explain below)				
Other 2 (please explain below)				
Other 3 (please explain below)				

17. Please describe the “other” challenges members of your team have encountered in engaging these patients and other stakeholders.
18. Please describe how you resolved the challenges identified above.
19. Do you plan to assess the level of influence of these patients and other stakeholders on your research project (s)?
  - Yes, please describe: *(Skip to Q 21)*
  - No
20. Would you be interested in assessing the level of influence of these patients and/or stakeholders on your research project (s)?
  - Yes
  - No

21. Up to this point, have you collected any information about the experiences of patients and other stakeholders engaged in your research project (s) (e.g., level of satisfaction with their role)?
  - Yes
  - No *(skip to Q 23)*
22. Please describe the type of information you have collected regarding the experiences of these patients and other stakeholders.
23. Do you have plans to collect information on the experiences of patient and other stakeholders engaged in this/these research project (s) in the future?
  - Yes
  - No
24. Please feel free to share any further comments or suggestions on engaging patients and other stakeholders in research.

Thank you for taking the time to complete this survey.

Your responses have been recorded.

On behalf of the CBPHC teams, we thank you for your contribution to helping us understand the nature, extent and experience of patient and stakeholder engagement in research processes.

## Abbreviations

CBPHC: Community-based primary health care
CIHR: Canadian Institutes of Health Research
NPI: Nominated principal investigator
PCORI: Patient-Centered Outcomes Research Institute
SPOR: Strategy for Patient Oriented Research
